# Contemporary trends for urological training and management of stress urinary incontinence in Ireland

**DOI:** 10.1007/s00192-021-04887-8

**Published:** 2021-06-23

**Authors:** Eoin MacCraith, James C. Forde, Fergal J. O’Brien, Niall F. Davis

**Affiliations:** 1 Royal College of Surgeons in Ireland, Tissue Engineering Research Group, Dublin, Ireland; 2 Department of Urology, Blackrock Clinic, Dublin, Ireland

**Keywords:** Stress urinary incontinence, Mesh, Sling, Urology, Gynaecology, Training

## Abstract

**Introduction and hypothesis:**

The aim of this study is to evaluate the trends in stress urinary incontinence (SUI) surgery since the 2018 pause on use of the polypropylene (PP) mid-urethral sling (MUS) and to quantify the effect this has had on surgical training.

**Methods:**

Two anonymous surveys were sent to all current urology trainees and to all consultant surgeons who specialise in stress urinary incontinence surgery.

**Results:**

Prior to the pause, 86% (6 out of 7) of consultant urologists and 73% (11 out of 15) of consultant gynaecologists would “always”/“often” perform MUS for SUI. After that, 100% (22 out of 22) of consultants reported that they “never” perform MUS. There has been a modest increase in the use of urethral bulking agent (UBA) procedures among urologists, with 43% (3 out of 7) now “often” performing this, compared with 71% (5 out of 7) “never” performing it pre-2018. Trainee exposure to SUI surgery reduced by 75% between 2016 and 2020. Despite a ten-fold increase in UBA procedures logged by trainees, the decline in MUS has resulted in a major reduction in total SUI surgeries. Coinciding with this decrease in surgeries, there was a 56% reduction in trainees’ self-assessed competence at SUI surgery. Thirteen percent of trainees are interested in specialising in Female Urology and those trainees had significantly greater exposure to SUI procedures during their training than those who did not (*p =* 0.0072).

**Conclusions:**

This study has identified a downward trend in SUI surgery, which is concerning for the undertreatment of females with SUI. A decline in SUI surgery training has resulted in reduced trainee confidence and interest in this subspecialty.

## Introduction

The use of polypropylene (PP) mesh for stress urinary incontinence (SUI) was banned in Australia in 2017 and in the UK and Ireland in 2018 [1, 2]. Since then, there has been a notable decrease in the overall treatment of SUI in Australia and the UK, despite some increase in the number of urethral bulking agent (UBA) procedures performed [3]. This may be due to patients being unsuitable to undergo traditional operations such as autologous fascial sling (AFS) and colposuspension or a lack of urologists and urogynaecologists trained in these procedures. Either way it leaves a significant proportion of the population with untreated debilitating SUI symptoms.

There are no data to date that demonstrate the scale of undertreatment of women with SUI in Ireland since the “pause” on PP mesh, or the effect this pause has had on surgical training. The primary aim of this study is to assess trends in surgical management for SUI in Ireland since the “pause” on PP mesh in 2018. The secondary aims are to assess their impact on surgical training and to pre-empt their effects on future workforce planning for female urology in Ireland.

## Materials and methods

### Participants

Institutional review board (IRB) approval was granted for this study (Research Ethics Committee reference REC202101012). Two separate anonymous online surveys were created using Google Documents©. The first survey (Survey A, Table 1) was distributed via email to all current urology trainees on the Higher Specialist Training (HST) pathway in Ireland. The second survey (Survey B, Table 2) was distributed via email to all consultant urologists and urogynaecologists in Ireland who specialise in stress urinary incontinence surgery. This cohort of consultants was reflective of all surgeons managing SUI in the jurisdiction. The SUI procedures listed in Survey A and Survey B were selected based on the urology curriculum for Irish trainees [4] and the EAU guidelines on urinary incontinence [5].

**Table 1:** Survey sent to urology trainees regarding their training in managing stress urinary incontinence (SUI)

**Question**	**Answer**
1. What year did you commence your Higher Specialist Training?	Free text
2. How many times have you assisted a mid-urethral sling?	Free text
3. How many times have you performed a mid-urethral sling?	Free text
4. How many times have you assisted an autologous fascial sling?	Free text
5. How many times have you performed an autologous fascial sling?	Free text
6. How many times have you assisted a colposuspension?	Free text
7. How many times have you performed a colposuspension?	Free text
8. How many times have you assisted injection of a urethral bulking agent?	Free text
9. How many times have you performed injection of a urethral bulking agent?	Free text
10. At the end of your training how competent do you feel you will be at performing surgery for SUI?	Multiple choice scale from 1 to 10
11. Prior to the mesh “pause” in 2018, please rank what would have been your order of preference for management of a healthy female patient with uncomplicated SUI with failed non-surgical treatment. (rank 1–5)	Mid-urethral sling
	Autologous fascial sling
	Colposuspension
	Urethral bulking agent
	Continue non-surgical management
12. Present day, please rank your order of preference for management of a healthy female patient with uncomplicated SUI with failed non-surgical treatment. (rank 1–5)	Autologous fascial sling
	Colposuspension
	Urethral bulking agent
	Continue non-surgical management
	Refer to a jurisdiction where mid-urethral sling is offered
13. If you selected “continue non-surgical management” in the previous question please select why, otherwise choose N/A.	Consultants in my department are not trained in these procedures
	Consultants in my department are trained in these procedures but do not perform them often enough and therefore prefer not to offer it
	I believe non-surgical management is superior to these treatments
	N/A
14. What area of urology do you hope to specialise in?	Stone disease
	Robotic and laparoscopic surgery
	Female pelvic medicine and reconstructive surgery
	Transplant
	Uro-oncology
	Andrology/male GU reconstruction and prosthetics
	Voiding dysfunction
	Paediatric urology
	General urology
	Unsure
15. Did you previously choose to specialise in Female Urology and change your mind following the mesh controversy?	Yes
	No

**Table 2:** Survey sent to consultant urologists and gynaecologists regarding their preferences for stress urinary incontinence (SUI) surgery before and after the “pause” on polypropylene in 2018

**Question**	**Answer**
1. I am a:	Urologist
	Gynaecologist
2. I have been a consultant for:	<5 years
	5–10 years
	>10 years
3. Prior to the “pause” on polypropylene mesh use for SUI, for a healthy female patient with uncomplicated SUI who has failed non-surgical treatment I would perform a:	Multiple choice for each operation:
	• Mid-urethral sling (never, rarely, sometimes, often, always)
	• Autologous fascial sling (never, rarely, sometimes, often, always)
	• Colposuspension (never, rarely, sometimes, often, always)
	• Urethral bulking agent (never, rarely, sometimes, often, always)
4. Since the “pause” on polypropylene mesh use for SUI, for a healthy female patient with uncomplicated SUI who has failed non-surgical treatment I would perform a:	Multiple choice for each operation:
	• Mid-urethral sling (never, rarely, sometimes, often, always)
	• Autologous fascial sling (never, rarely, sometimes, often, always)
	• Colposuspension (never, rarely, sometimes, often, always)
	• Urethral bulking agent (never, rarely, sometimes, often, always)

### Data collection

Survey A consisted of 15 questions (Table 1) relating to the year each trainee’s HST training commenced, operative exposure to a mid-urethral sling (MUS), AFS, colposuspension and UBA, self-rated SUI surgery competency, procedure preference for managing SUI, and finally chosen urological subspecialty interest.

Survey B consisted of four questions (Table 2) relating to surgical specialty (i.e. urologist or urogynaecologist), duration of consultant status, frequency of performing MUS, AFS, colposuspension and UBA prior to and after the 2018 “pause” on PP mesh. Urologists and urogynaecologists were questioned on the frequency of surgical treatments performed for uncomplicated SUI following failed non-surgical management. Respondents reported on the frequency with which they performed various SUI procedures before and after 2018 on an ordinal scale (never, rarely, sometimes, often, always).

### Data and statistical analysis

Continuous variables are presented as mean ± standard deviation and Student’s *t* test was used for pairwise comparisons. Categorical data are presented as number (percentage [%]) and Fisher’s exact test was used for comparisons. Statistical analysis was performed using GraphPad (La Jolla, CA, USA) and significance was set at *p* < 0.05.

## Results

### Consultant respondents

**Fig. 1:**
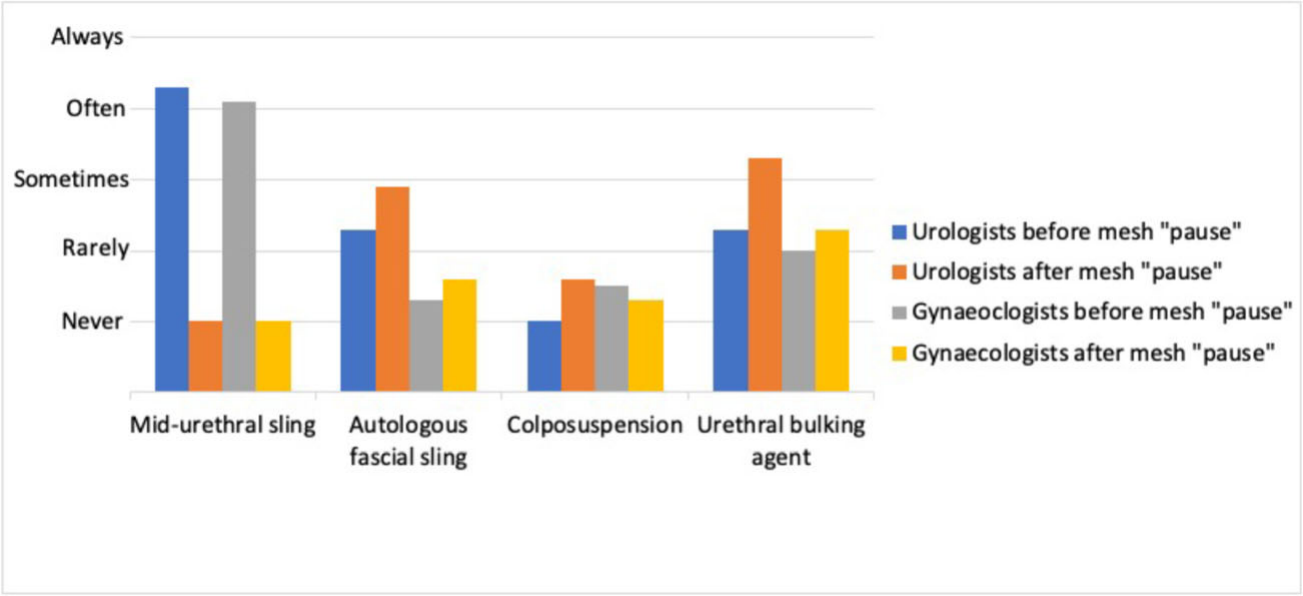
Trends in stress urinary incontinence operations performed before and after the “pause” on the use of mesh in 2018: a comparison of urologists and gynaecologists. This graph illustrates the dramatic reduction in midurethral sling surgery, which is equal among urologists and gynaecologists. There has been a greater increase in alternative procedures such as urethral bulking agent and autologous fascial sling among urologists compared with gynaecologists

Twenty-two (88%) consultants responded to the survey invitation. Seven respondents were primarily urologists and 15 were urogynaecologists. At the time of survey completion 14 (63.6%) respondents had been qualified consultants for >10 years, 4 (18.2%) had been consultants for 5–10 years and 4 (18.2%) had been consultants for <5 years. As illustrated in Fig. 1, the overall use of MUS has decreased from “often” to “never”. UBA increased from “rarely” to “sometimes” and this rise has been more notable with urologists. AFS has increased from “never” to “rarely” and is also more notable with urologists. There has been no change in colposuspension frequency.

Prior to the pause, 43% (3 out of 7) of urologists said they “always” performed MUS and 43% (3 out of 7) said they “often” performed MUS. At that time, 71% (5 out of 7) said they “rarely” performed UBA. After 2018, 100% of urologists said they “never” do MUS anymore and 43% (3 out of 7) say they now do UBA “often”. For urogynaecologists, prior to the pause, 40% (6 out of 15) reported that they did MUS “often” and 33% (5 out of 15) “always” performed MUS for SUI. At that time, 40% (6 out of 15) said that they “rarely” and 33% (5 out of 15) said that they “never” used a UBA. After the pause, 100% (15 out of 15) said that they “never” perform MUS, and 27% (4 out of 15) report that they now “often” use a UBA; however, 40% (6 out of 15) “never” use a UBA. These trends are illustrated in Fig. 1.

### Trainee respondents

Twenty-three (88%) trainees responded to the survey invitation. Trainee grade ranged from year 1 to year 6 and the survey included respondents from all years. Trainees logged 6 SUI procedures/trainee/year over the last 7 years of surgical training in Ireland. Records peaked at 14.13 in 2016 and this is followed by a steady decline (i.e., 75%) to 3.5 procedures/trainee/year in 2020.

**Fig. 2:**
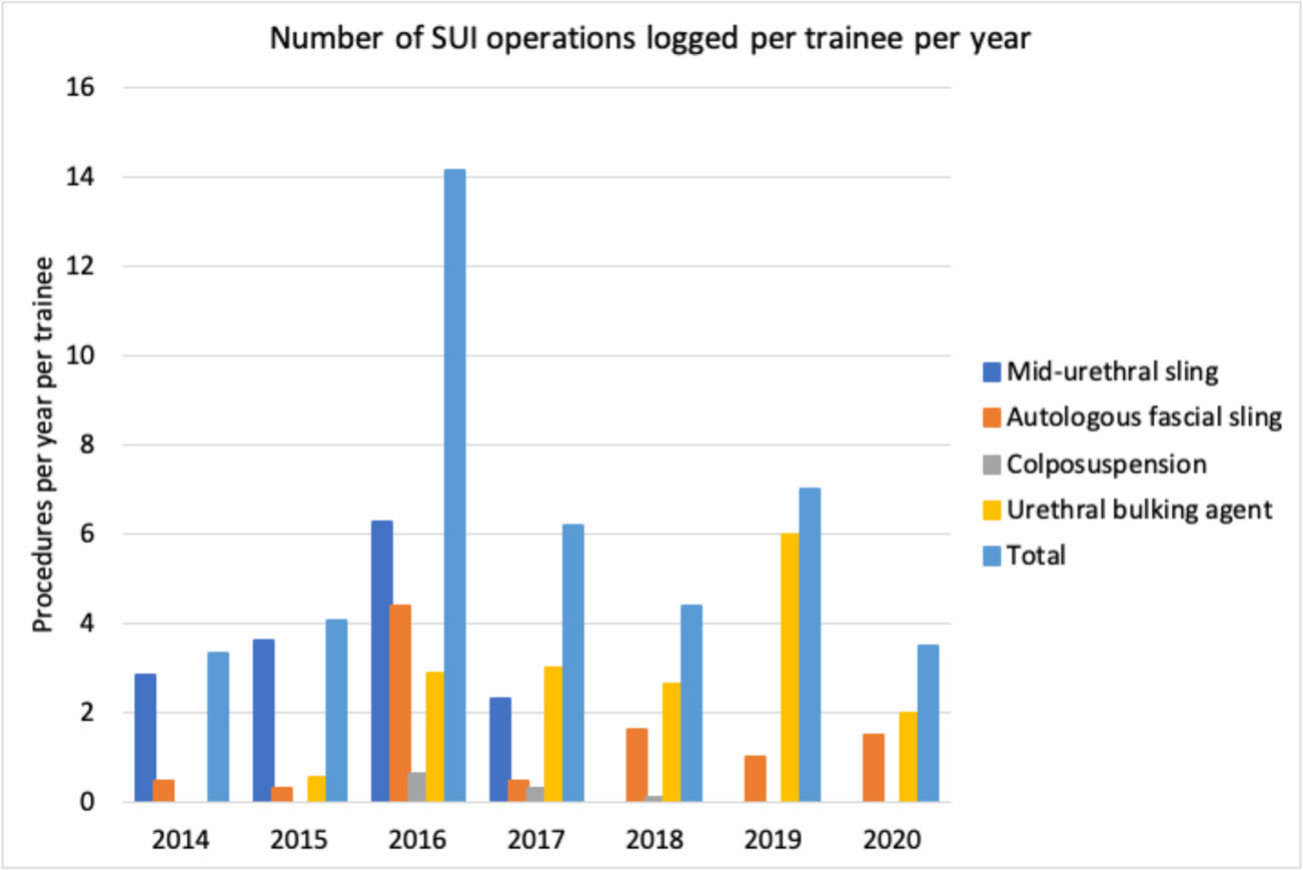
Annual number of stress urinary incontinence (SUI) operations logged by trainees per year. This chart illustrates the decline in SUI operations logged by urology trainees since 2014. Midurethral sling has dramatically declined whereas urethral bulking agent and autologous fascial sling have increased

The MUS was the most common SUI operation logged by trainees before 2018. It peaked at 6.25 procedures/trainee/year in 2016 and decreased by 63% to 2.33 in 2017. No MUS procedures have been logged by trainees since 2017. UBA procedures demonstrated a steady increase since 2015 (except for 2020, which can be explained by operative limitations due to the COVID-19 global pandemic). From 2015 to 2019, there was a 10-fold increase in UBA (from 0.56 to 6) procedures/trainee/year. AFS peaked at 4.38 procedures/trainee/year for 2016 trainees, but has been in decline since then, averaging 1.16 procedures/trainee/year. Colposuspension procedures have remained lower over the past 7 years, averaging 0.15 procedures/trainee/year. These trends are illustrated in Fig. 2.

#### Trainee subspecialty preference

Thirteen percent of trainees plan to subspecialise in the field of female pelvic medicine and reconstructive surgery (FPMRS). Trainees who developed an interest in FPMRS were exposed to a significantly greater number of SUI procedures during their training (39.33 ± 35.02 versus 12.25 ± 10.46, *p* = 0.0072).

#### Trainee competence

**Fig. 3:**
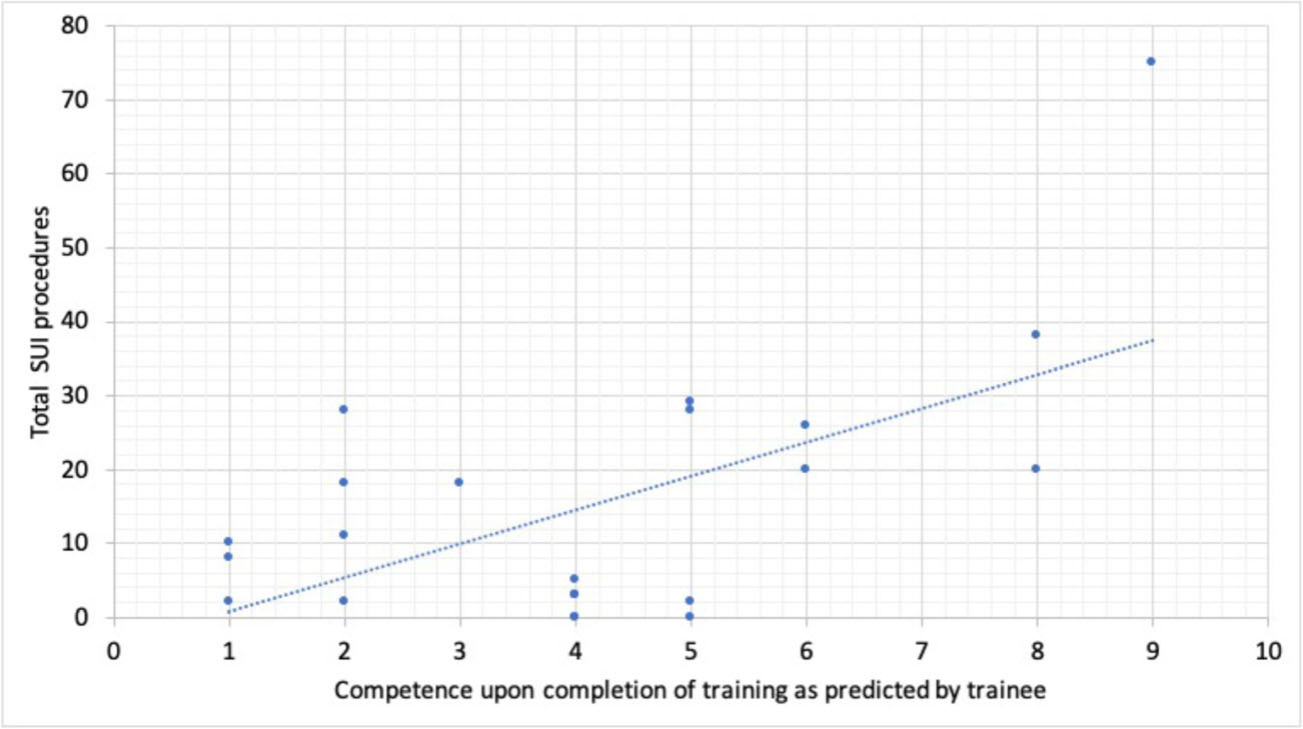
Correlation between number of stress urinary incontinence (SUI) procedures logged by trainees and trainees self-assessed competence. This graph illustrates that trainees who were exposed to more SUI operations during training had greater confidence that they would be competent in this field by the time they had completed their training scheme

Trainees were questioned on competency on a scale of 1–10 for SUI surgery upon completion of their surgical training based on their surgical exposure. Predicted competence peaked at 8.5 among 2016 trainees coinciding with the greatest number of SUI procedures and MUS surgery. Since 2018, the average self-rating score was 3.7 and this corresponds to a 56% reduction in competence. Figure 3 illustrates the relationship between the number of SUI procedures to which trainees are exposed and their self-rated predicted competence at conclusion of training.

#### Trainee treatment preference

Trainees were questioned on preferred treatment options for uncomplicated SUI following failed non-surgical management before and after 2018. MUS decreased from 74% to 0% (*p* < 0.0001). UBA increased from 9% to 35% (*p* = 0.035). AFS increased from 0% to 35% (*p* = 0.002). Colposuspension decreased from 13% to 4% (*p* = 0.279). Non-surgical management increased from 4% to 17% (*p* = 0.155). Referring the patient to another jurisdiction for MUS increased from 0% to 9% (*p* = 0.145).

## Discussion

To our knowledge, this is the first Irish study to evaluate the downward trend in SUI surgery since the “pause” on the use of polypropylene mesh for MUS in 2018. We demonstrate that consultant surgeons, predominantly urologists rather than urogynaecologists, have only modestly increased the use of UBA and AFS surgery. We also found a concerning reduction in trainees’ exposure to SUI surgery annually since 2016 and only 13% of trainees plan on specialising in the FPMRS subspecialty. Finally, trainee confidence and interest in the subspecialty is decreasing and is related to the procedure exposure rate.

One recent study has shown that there has been a significant decline in both MUS surgery and total SUI procedures performed in the public sector in Australia and England in the last 10 years [3]. Our findings also show a similar decline in MUS and SUI surgery, with a modest increase in UBA procedures. Similar data from 2020 show that the use of MUS declined by 50% between 2011 and 2013 in the USA, as well as an overall decline in SUI surgery [6]. In Ireland, the aim is for ≥1 FPMRS urologist to resource each of the six hospital groups [7]. Our study provides contemporary evidence that it will be challenging to meet this goal, as only three trainees have declared an interest in this field. We also demonstrate a concerning decline in SUI surgery logged by trainees over the last 7 years. These findings indicate a potential future healthcare staffing issue that should be addressed by attempting to foster interest in the subspecialty among urological trainees.

The current urology curriculum also needs to be capable of adapting to the findings highlighted in the present study. The 2021 urology curriculum in Ireland and the UK requires that trainees log 9 MUS, 9 UBA and 9 AFS or colposuspensions at skill level II (able and trusted to act with direct supervision) by completion of HST [4]. However, we demonstrate that only a minority of trainees will meet these requirements. To remedy this, one suggestion is that Irish surgeons who are fellowship-trained in FPMRS could increase the number of alternative procedures being performed; however, this suggestion is also not without limitations. For example, colposuspension has a longer duration of inpatient stay and a higher incidence of associated pelvic organ prolapse (POP) [8]. Autologous slings have higher rates of urinary retention, wound complications and longer operative duration [9]. Urethral bulking agents have lower cure rates than MUS [9].

One limitation of the present study is that it may not accurately reflect the true number of procedures being performed in Irish hospitals as it relies somewhat on the reliability of trainee logbooks and consultant recall while completing the survey. Despite this, it is a comprehensive survey completed by 88% of consultants and trainees from all HST grades in Ireland and serves as a useful contemporary tool that can guide policy makers in terms of quality improvement in the specialty.

## Conclusion

We have identified two concerning findings for urologists and urogynaecologists. The first is a decline in the rate of SUI surgery reported by consultant surgeons, which is worrying for undertreatment of women with SUI. The second finding is a downward trend from 2013 to 2020 in the exposure of surgical trainees to SUI procedures, with a resultant decreased interest in Female Urology that will lead to challenges in meeting staffing targets in the future.

## Abbreviations

AFS: Autologous fascial sling
FPMRS: Female pelvic medicine and reconstructive surgery
HST: Higher specialist training
MUS: Mid-urethral sling
PP: Polypropylene
SUI: Stress urinary incontinence
UBA: Urethral bulking agent

## References

[1] Ugianskiene A, Davila GW, Su TH (2019). FIGO review of statements on use of synthetic mesh for pelvic organ prolapse and stress urinary incontinence. Int J Gynaecol Obstet.

[2] Department of Health, Ireland (2018) Minister for Health Simon Harris announces pause in the use of transvaginal mesh devices. Available from: https://www.gov.ie/en/press-release/6c85c7-minister-for-health-simon-harris-announces-pause-in-the-use-of-trans/. Accessed 1 Mar 2021

[3] Mathieson R, Kippen R, Manning T, Brennan J (2020). Stress urinary incontinence in the mesh complication era: current Australian trends. BJU Int.

[4] ISCP, Intercollegiate Surgical Curriculum Programme. Urology Curriculum. 2021. Available from: www.iscp.ac.uk Accessed 2nd March 2021.

[5] Burkhard FC, Bosch JLHR, Cruz F. EAU Guidelines on Urinary Incontinence in Adults 2018, in European Association of Urology Guidelines. 2018 Edition. European Association of Urology Guidelines Office: Arnhem, The Netherlands.

[6] Lee UJ, Feinstein L, Ward JB (2020). National trends in the surgical management of urinary incontinence among insured women, 2004 to 2013: the Urologic Diseases in America Project. J Urol.

[7] Rogers E, Lonergan P. Urology: a model of care for Ireland. 2019. Available from: https://www.rcsi.com/surgery/-/media/feature/media/download-document/surgery/practice/publications-and-guidelines/models-of-care/urology--a-model-of-care-for-ireland.pdf. Accessed 23 December 2020

[8] Rehman H, Bezerra CA, Bruschini H, Cody JD, Aluko P (2017). Traditional suburethral sling operations for urinary incontinence in women. Cochrane Database Syst Rev.

[9] Kirchin V, Page T, Keegan PE, Atiemo K, Cody JD, McClinton S. Urethral injection therapy for urinary incontinence in women. Cochrane Database Syst Rev. 2012;(2):CD003881. 10.1002/14651858.CD003881.pub3 22336797

